# Clinical features, risk factors, and outcomes of neonatal invasive candidiasis: a 20-case study series from a tertiary neonatal critical care unit in Oman

**DOI:** 10.1186/s13052-025-01952-6

**Published:** 2025-05-22

**Authors:** Basem Abdelhadi, Mujtaba Al Ajmi, Hilal Al Hashami, Manal Al Rawahi

**Affiliations:** 1 Child Health Department, Royal Hospital, Muscat, Sultanate of Oman; 2 Child Health Department, Pediatric Infectious Diseases Consultant, Royal Hospital, Muscat, Sultanate of Oman; 3 Child Health Department, Nizwa Hospital, Nizwa, Sultanate of Oman

## Abstract

**Background:**

One of the well-known causes of morbidity and mortality in the neonatal population is invasive candidiasis. In neonatal critical care units, candida sepsis is now the third most frequent cause of late-onset sepsis.

The objective of this research was to determine the prevalence of related risk factors, clinical traits, and results related to invasive candidiasis in the neonatal intensive care unit (NICU) at the Royal Hospital in Oman.

**Methods:**

A case–control retrospective analysis of 20 newborns admitted between January 2006 and December 2020 to the neonatal intensive care unit at Royal Hospital. In terms of gestational age, birth weight, and date of birth, cases and controls were matched.

**Results:**

There were 60 infants in all- 20 cases and 40 controls. The two groups'demographics, including age, sex, and weight, were comparable. The case group compared to the control group had a mean gestational age of 32.2 ± 6.1 weeks against 32.2 ± 5.7 weeks and a mean birth weight of 1978 ± 1202 grammes against 1930 ± 1040 grammes, respectively. A total of 14,820 newborns were admitted to the NICU during the study period, with 20 cases of candidemia, representing an incidence of approximately 1.3 per 1000 admissions. Seventy-five percent of the isolated species were Candida albicans. Sixty-five percent of the instances happened in the second and third week of life. Compared to the control group, the case group's mean NICU stay was longer. Several risk factors, including young maternal age, the presence of a central line, invasive mechanical ventilation, total parenteral nutrition, prolonged hospital stay, necrotizing enterocolitis, abdominal surgeries for congenial intestinal malformations and cardiac surgeries for congenital heart diseases, were found to be related with an elevated risk for invasive candidiasis using univariate analysis. In the case group, the overall mortality rate was 45%, but in the control group, there was no death in the cohort.

**Conclusion:**

The following factors were linked to an elevated risk of invasive candidiasis in this case series: total parenteral nutrition, prolonged hospital stay, central line presence, prematurity, abdominal and cardiac surgeries. Those with risk characteristics should be highly suspected for neonatal candidiasis, particularly if their stay in the NICU is longer than seven days. Antifungal prophylaxis should be taken into consideration for these newborns, and presumed antifungal medication should be started as soon as possible.

## Introduction

One of the well-known causes of morbidity and mortality in the neonatal population is invasive candidiasis [1, 2]. Following Gram-negative bacteria and Coagulase-negative Staphylococci as the two most frequent causes of late-onset sepsis in neonatal critical care units, Candida sepsis has emerged as the third most frequent cause [3, 4]. Until recently, Candida albicans was the most often isolated and invasive species that spread disease widely. On the other hand, non-albicans Candida was becoming more frequently identified as the cause of late-onset newborn sepsis [5–7]. 20% of newborns with invasive candidiasis die from it, and this percentage might rise to 30% in babies with particularly low birth weights [8], and severe neurodevelopmental damage affects 50% of survivors [9]. End-organ damage is also frequently observed in the heart, genitourinary tract, and central nervous system [10]. Low birth weight, premature birth, central catheterization, days of artificial breathing, abdominal surgery, exposure to broad spectrum antibiotics, usage of H2 blockers, and steroids are risk factors for invasive neonatal candidiasis that have been identified [11].

## Methodology

This study assessed a group of twenty neonates who were diagnosed with invasive candidiasis and were admitted to Royal Hospital (RH), Oman, retrospectively. With 40 beds available, the Royal Hospital's neonatal intensive care unit (NICU) is Oman's largest tertiary NICU. An isolation of Candida species from a sterile location (blood) was considered a diagnosis of invasive candidiasis in that particular case. Infants having endotracheal tube cultures or urine cultures that were just positive were excluded. A matching pair of patients served as controls for every instance of invasive candidiasis.

Utilising gestational age, birth weight category ("extremely low birth weight (ELBW) < 1000 grammes, very low birth weight (VLBW) 1001–1500 grammes, low birth weight (LBW) 1501–2500 grammes, and > 2500 grammes"), and birth date, control infants were matched to cases.

In order to determine the effect of invasive candidiasis on clinical outcomes, such as NICU length of stay and all-cause death, our study adjusted for gestational age. Given that multiple studies have already found a high correlation between ELBW and VLBW and an increased risk of developing invasive candidiasis, birth weight was regulated [6, 8]. To guarantee that the treating agents and the infecting Candida species were similar, birth dates were matched for both the case and control groups.

Data on cases and controls were taken from the neonatal admission record at the Royal Hospital. The Royal Hospital's Al-Shifa 3 + hospital electronic information system provided the comprehensive clinical, laboratory, and demographic data on the infants. Every episode's research period included the period from hospital admission to discharge or death.

Maternal age (GA), gender, birth weight, age at infection, central line, endotracheal tube (ETT), invasive ventilation days, total parenteral nutrition (TPN), TPN days, length of stay, type of surgery (if any), use of H2 blocker, use of dexamethasone and duration, candida species, susceptibility to antifungal agents, and thrombocytopenia (defined as a platelet count below 150,000/µL), were among the neonatal data that were collected.

Premature rupture of membranes (PROM), high vaginal swab (HVS) culture screening for candida, birth method, perinatal antibiotics, and antenatal steroids were the variables. The following were the long-term effects: death, cerebral palsy, blindness, hearing loss, and cognitive decline.

### Statistical analysis

The case and control groups'demographics, risk factors, and outcome characteristics were contrasted. Mann–Whitney for continuous variables (such as age, length of hospital stay, and duration of antibiotic medication), the U rank-sum test and the t-test were employed. For categorical variables (such as gender, use of broad-spectrum antibiotics, infecting organism, and death), the chi-square test and Fisher's exact test were employed. Two-tailed analyses were used for all statistical tests.

When *p-values* were less than 0.05, statistical differences between the case and control groups were deemed significant. We performed univariate analysis on both continuous and nominal variables. A multivariable logistic regression model was utilized to evaluate potential independent variables that yielded *p-values* ≤ 0.05 in the univariate studies. Analysis was done both backwards and forwards stepwise to account for confounding variables when determining independent risk factors linked to the emergence of newborn candidiasis. An independent statistician used SPSS, version 20 (SPSS Inc., Chicago, USA) for all statistical analyses.

## Results

The study comprised 60 infants in total (20 cases and 40 controls), all of whom were admitted to the RH NICU between January 2006 and December 2020. A total of 14,820 newborns were admitted to the NICU during the study period. The incidence of candidemia was determined to be approximately 1.3 per 1,000 admissions, corresponding to 20 identified cases. Regarding gestational age and birth weight, there were no notable variations. Table 1 shows that the case group's mean gestational age was 32.2 ± 6.1 weeks compared to 32.2 ± 5.7 weeks, and its mean birth weight was 1978 ± 1202 grammes compared to 1930 ± 1040 grammes. Six of the sixteen newborns with ELBW (cases and controls) experienced invasive candidiasis. Four of the twelve newborns with VLBW-both cases and controls-also experienced invasive candidiasis. In the case group, the overall mortality rate was 45%, but in the control group, there was no death at all.

**Table 1:** Demographic characteristics in cases and controls

Characteristic	Case (*n* = 20)	Control (*n* = 40)	*p*-value
Gestational age in weeks—Mean ± SD	**32.25 ± 6.12**	**32.28 ± 5.74**	**0.988**
Sex			
Male—no (%)	**15 (75)**	**18 (45)**	
Female—no (%)	**5 (25)**	**22 (55)**	
Birth weight (grams)			
Mean ± SD	**1978 ± 1202**	**1930 ± 1040**	**0.913**
Mode of delivery			
SVD—no (%)	**16 (80)**	**22 (55)**	
LSCS—no (%)	**4 (20)**	**18 (45)**	
Length of stay (days) -			
Mean ± SD	**52.95 ± 43.49**	**31.58 ± 31.16**	**0.041**

There were 15 male infants (75%) and 5 female infants (25%) in the case group, while there were 18 male infants (45%) and 22 female infants (55%) in the control group (Table 1). The median length of stay and median age before candidemia were 52.95 (range, 3 to174) and 16.8 (range, 4 to 57) days, respectively. More than half of the cases (65%) occurred during the second and third week of life; only 3 infants were < 7 days of age when invasive candidiasis occurred and 3 infants were > 30 days at 31,32 and 57 days of life.

**Fig. 1:**
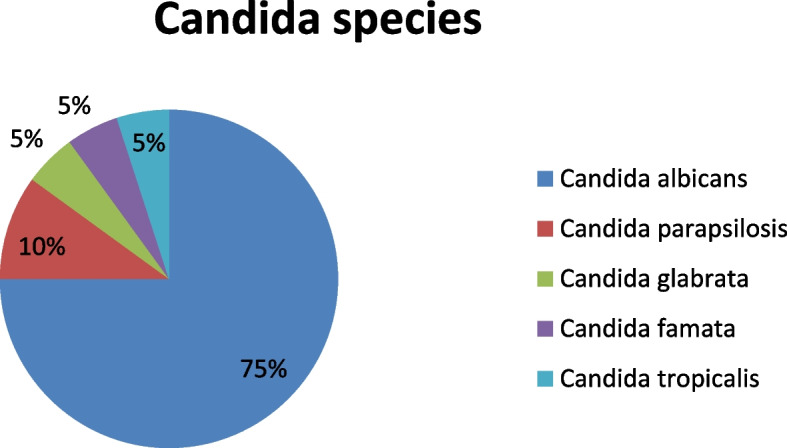
Analysis of Candida species

Analysis of Candida species:

In this cohort, 75% (*n* = 15) of instances of invasive candidiasis were caused by* Candida albicans.* (Fig. 1), followed by *Candida parapsilosis* causing 10% (*n* = 2). Other Candida species were *Candida glabrata*, causing 5% (*n* = 1), *Candida tropicalis*, causing 5% (*n* = 1) and *Candida famata*, causing 5% (*n* = 1). The characteristics of fungal infection and duration of anti-fungal therapy are shown in Table 4.

### Risk factors

Factors that were shown to be substantially linked with candidemia in the univariate logistic regression studies can be categorised as follows:

Maternal risk factors.

The study showed a significant risk factor of young maternal age (*p* = 0.034) with mean age of 26.8 years. There were no significant risk factors of the followings: perinatal antibiotics, steroid use, PROM, HVS and mode of delivery (Table 2).

**Table 2:** Maternal characteristics of the case group

	**Maternal age (years)**	**Premature rupture of membranes (PROM)**	**High vaginal swab (HVS) positive for Candida**	**Mode of delivery**	**Perinatal antibiotics**	**Antenatal steroids**
1	22	No	No	SVD	Yes	No
2	24	No	unknown	SVD	unknown	No
3	26	No	No	SVD	No	No
4	-	Unknown	unknown	SVD	No	No
5	-	Unknown	unknown	SVD	No	No
6	-	Unknown	unknown	SVD	unknown	No
7	-	Unknown	unknown	SVD	unknown	No
8	-	Unknown	unknown	SVD	unknown	No
9	24	No	No	SVD	No	No
10	-	Unknown	unknown	SVD	No	Yes
11	34	No	No	SVD	No	No
12	-	Unknown	unknown	LSCS	No	Yes
13	24	No	No	SVD	No	No
14	28	No	No	LSCS	No	Yes
15	29	Unknown	unknown	SVD	No	Yes
16	-	Unknown	unknown	LSCS	unknown	unknown
17	36	No	unknown	SVD	No	No
18	21	No	Yes	SVD	Yes	Yes
19	-	Unknown	unknown	LSCS	unknown	Unknown
20	-	Unknown	unknown	SVD	unknown	No

#### Central lines

The presence of central line catheterization was significantly associated with invasive candidiasis (*p* < 0.001) with mean duration of 12.65 days, endotracheal tube (*p* = 0.006) with mean duration of 17.85 days, and total paternal nutrition (*p* < 0.001) with mean duration of 20.35 days (Table 3).

**Table 3:** Risk factors in cases and controls

Risk factor	Case (*n* = 20)	Control (*n* = 40)	*p*-value
Central line—no (%)	**17 (85)**	**14 (35)**	
Central line days -			
Mean ± SD	**12.65 ± 10.58**	**2.75 ± 3.93**	**< 0.001**
Endotracheal tube—no (%)	**17 (85)**	**19 (47.5)**	
Invasive ventilation days -			
Mean ± SD	**17.85 ± 19.33**	**2.53 ± 4.86**	**< 0.001**
Total parenteral nutrition			
(TPN)—no (%)	**18 (50)**	**18 (50)**	
TPN days—Mean ± SD	**20.35 ± 18.30**	**4 ± 5.2**	**< 0.001**
Surgery—no (%)	**12 (60)**	**0 (0)**	
Duration of H2 blocker use			
(days)—Mean ± SD	**5 ± 10.93**	**0 ± 0**	**< 0.001**
Duration of dexamethasone use			
(days)—Mean ± SD	**0.4 ± 1.79**	**0 ± 0**	**0.157**
Candida colonization before			
infection—no (%)	**8 (40)**	**0 (0)**	**< 0.001**

#### Others

Other risk variables, such as necrotizing enterocolitis (*p* = 0.01), surgery (*p* < 0.001), and Candida colonisation in urine or sputum (*p* < 0.001), were also strongly linked to invasive candidiasis in newborns. Among the cases of invasive candidiasis, abdominal surgeries were performed in 6 cases for conditions such as intestinal malrotation (3 cases), meconium ileus (2 cases), and duodenal atresia (1 case), followed by cardiac surgeries in 4 cases for congenital heart diseases including transposition of the great arteries (3 cases) and total anomalous pulmonary venous drainage (1 case). Other risk factors include: prolonged hospital stay (*p* = 0.041), use of H2 blockers (*p* < 0.001). There was no risk factor of dexamethasone use (*p* = 0.157). Thrombocytopenia, defined as a platelet count below 150,000/µL, was strongly associated with the case group (*p* < 0.001), see Table 3.

#### Outcome

**Fig. 2:**
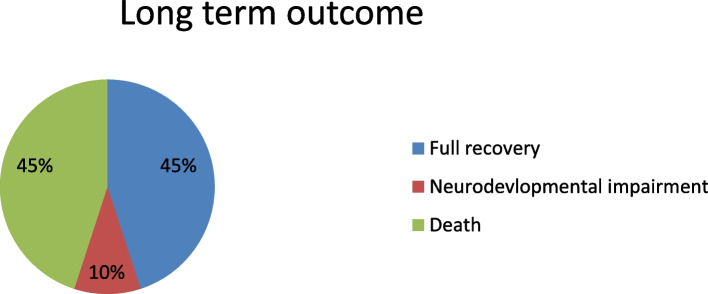
Long term outcome of invasive candidiasis

The following three outcomes were used to measure the long-term outcome: death, neurodevelopmental disability, or full recovery. Figure 2 shows that 45% of patients had a full recovery, 10% had a recovery with complications, and 45% had died.

#### Treatment response and resistance profile

16 cases were treated with liposomal amphotericin B, while 4 cases received fluconazole. The treatment response for both liposomal amphotericin B and fluconazole was favorable in 55% of cases, with no reported resistance to antifungal agents, see Table 4.

**Table 4:** Characteristics of fungal infection

	**Candida species**	**Site of isolation**	**Susceptibili-y to antifungal agents**	**Antifungal agent used**	**Duration of treatment (days)**
1	*Albicans*	Blood	Sensitive	liposomal amphotericin B	4
2	*Albicans*	Blood	Sensitive	liposomal amphotericin B	21
3	*Albicans*	Blood	Sensitive	liposomal amphotericin B	14
4	*Albicans*	Blood	Sensitive	liposomal amphotericin B	3
5	*Albicans*	Blood	Sensitive	liposomal amphotericin B	25
6	*Albicans*	Blood	Sensitive	liposomal amphotericin B	14
7	*Albicans*	Blood	Sensitive	liposomal amphotericin B	8
8	*Albicans*	Blood	Sensitive	liposomal amphotericin B	42
9	*Albicans*	Blood	Sensitive	liposomal amphotericin B	65
10	*Albicans*	Blood	Sensitive	liposomal amphotericin B	38
11	*Tropicalis*	Blood	Sensitive	liposomal amphotericin B	31
12	*Glabrata*	Blood	Sensitive	liposomal amphotericin B	14
13	*Albicans*	Blood	Sensitive	Fluconazole	56
14	*Albicans*	Blood	Sensitive	Fluconazole	14
15	*Parapsilosis*	Blood	Sensitive	liposomal amphotericin B	17
16	*Famata*	Blood	Sensitive	liposomal amphotericin B	21
17	*Albicans*	Blood	Sensitive	Fluconazole	50
18	*Albicans*	Blood	Sensitive	liposomal amphotericin B	2
19	*Parapsilosis*	Blood	Sensitive	liposomal amphotericin B	1
20	*Albicans*	Blood	Sensitive	Fluconazole	34

## Discussion

By examining 20 cases of invasive candidiasis and contrasting them with the control group, this study sought to determine the clinical features, most prevalent related risk factors, and outcome of invasive candidiasis in the NICU at RH, Oman. These findings suggest that invasive neonatal candidiasis poses a significant risk to neonates receiving care in intensive care units. Male neonates made up the majority of those with invasive candidiasis in this study, accounting for 75% of the episodes. Some investigations conducted internationally showed a small male predominance [12, 13].

This study showed that invasive candidiasis linked inversely with gestational age and was highest in newborns with extremely low birth weight relative to low birth weight. The presence of risk factors, such as prematurity and low birth weight in neonates, was also correlated with this disease.

The finding that the more than half of the infections (65%) occurred after second week of life clearly indicated that this could be a complication of procedures and management of other medical and surgical conditions. This group of patients could have such as the need for TPN, abdominal surgeries for congenital intestinal malformations and cardiac surgeries for congenital heart diseases, NEC, presence of central lines and being on broad spectrum antibiotics. It also could represent a potential hospital acquired infection, and these findings are supported by other studies [14, 15].

International studies support the finding that Candida albicans was the most often isolated species of candida (75%). The various geographic regions had varying effects on the distribution of Candida species. With percentages ranging from 74 to 100% in Europe and from 40 to 69.2% in North and South America, Candida albicans was the predominant species in both regions. In Asia, the prevalence of Candida non-albicans species ranged from 25 to 92%, with a median of 75%. Candida non-albicans and albicans were similarly distributed in Australia (42% and 43%, respectively) [8, 16].

The results showed no significant correlation between perinatal antibiotics, steroid use, premature rupture of membranes, high vaginal swab culture screening for candida, mode of delivery and the subsequent development of candida infection in neonates. None of the patients were on antifungal prophylaxis during the study period.

Furthermore, this analysis supported the theory that the longer the NICU admission, use of broad-spectrum antibiotics, presence of central lines, use of H2 blockers, intestinal diseases and the higher the risk for a neonate to develop invasive candidiasis [17].

The results of this study confirmed the risk factors that were addressed and published internationally. These important findings should be studied and practical preventative strategies should be applied in the neonatal intensive care unit to reduce the risk to such high risk patients through guidelines and protocols of infection prevention and control and antimicrobial stewardship program.

The researchers examined three possible overall outcomes: death, full recovery, and recovery with neurodevelopmental disability. The death rate and neurological consequences are significantly higher, even with effective therapy [13]. More than 50% of survivors with neonatal invasive candidiasis had neurodevelopmental damage, according to the majority of studies; in other reports, this percentage even reached 75% [1, 18]. On the other hand, a research conducted in Saudi Arabia found that 33% of people died [13]. In another study, invasive candidiasis may be the cause of up to 50% of NICU patient deaths even with proper antifungal medication [18]. In our investigation, full recovery was reported to be 45%, recovery with sequelae to be 10%, and mortality to be 45%. In order to save this vulnerable group of newborns, it is a major discovery to address all relevant risk factors and preventative factors.

### Limitations of the study

Due to the retrospective nature of the study and the small number of invasive candidiasis cases it covered, the results'generalizability is restricted. The results cannot confirm that all kinds of antifungal drugs can affect candida species because there is insufficient data available. To determine the exact burden of invasive infections in high-risk newborns in our tertiary neonatal critical care unit, a more prospective research is needed.

## Conclusion

Our investigation for the risk variables at our hospital was motivated by the relevance of morbidity and death of newborn invasive candidiasis appearing as late-onset sepsis in critically unwell neonates. The following risk variables were demonstrated in this case series to be linked to an increased risk of invasive candidiasis: total parenteral nutrition, central line presence, prolonged hospital stay, prematurity, abdominal and cardiac surgeries. Newborns with these risk factors should be highly suspected of having neonatal candidiasis, particularly if their stay in the NICU exceeds seven days. Antifungal prophylaxis should be taken into consideration for these newborns, and presumed antifungal medication should be started as soon as possible.

Furthermore, only neonates weighing less than 1000 grammes are currently advised to get antifungal prophylaxis, according to the Infectious Diseases Society of America. The utilisation of risk factors found in this study suggests more infants who may benefit from prophylactic fluconazole administration; nevertheless, additional research is required to fully understand the advantages against dangers.

## Data Availability

The analyzed data sets available from the corresponding author on reasonable request after approval of the hospital research committee.
